# *BGIG10116_34868*: A Newly Discovered Gene Regulating Ejaculation Function

**DOI:** 10.3389/fphys.2022.762272

**Published:** 2022-02-28

**Authors:** Jingjing Gao, Rui Gao, Hu Li, Xi Liu, Pan Gao, Junhua Du, Hui Jiang, Xiansheng Zhang

**Affiliations:** ^1^Department of Urology and Andrology, The First Affiliated Hospital of Anhui Medical University, Hefei, China; ^2^Department of Andrology, The Third Hospital of Peking University, Beijing, China

**Keywords:** ejaculation function, rat, serotonin transporter, lifelong premature ejaculation (lifelong PE), genes

## Abstract

Ejaculation is a complex biphasic process involving a series of neurophysiological activities, such as the contraction of a large number of muscle groups and the ejaculation of semen from the urethra anterior. Due to the complexity of the process, many related factors have not been fully clarified, resulting in ejaculation dysfunction. As a common ejaculation dysfunction, lifelong premature ejaculation (LPE) is a problem for many people. Notably, gene polymorphism might play an important role in the etiology of LPE. However, the quest for identifying the actual genetic loci that contribute to LPE etiology has not been successful. Due to discrepancies in the design and methods of research, the correlation of most reports was not obtained in subjective replication experiments, and the conclusions may be inconsistent. In our study, three groups of ejaculation rats, namely, “rapid, normal, and delayed,” were selected based on the animal model of premature ejaculation (PE) in rats and the theory of ejaculation. Among them, the rats in the “rapid” ejaculation group can be used to stimulate humans with PE. Subsequently, we used the rat brain tissue for whole-transcriptome sequencing to screen the differential genes among the three groups. We tried to identify the actual genetic loci that contribute to PE etiology and provide a theoretical basis for the targeted therapy of PE.

## Introduction

Ejaculation is a complex biphasic process involving a series of neurophysiological activities, such as the contraction of a large number of muscle groups and the ejaculation of the semen from the anterior urethra (Alwaal et al., 2015). Three consecutive stages (including emission, expulsion, and orgasm) might constitute the process of ejaculation, and any problem in these stages would lead to ejaculation dysfunction (Forbes et al., 2018), such as premature ejaculation (PE) and delayed ejaculation. PE is a prevalent male ejaculation dysfunction with epidemic data showing that 20–30% of adult men are affected (Rowland, 2011; El-Hamd et al., 2019). Based on the recent definition of the International Society of Sexual Medicine (ISSM), PE is divided into two categories: lifelong PE (LPE) and acquired PE (APE) (Serefoglu et al., 2014). LPE has the following three characteristics: a) ejaculation has always occurred prior to or within about 1 min of vaginal penetration; b) those who are always or almost unable to control delayed ejaculation; and c) those who have negative personal consequences, such as distress, bother, and/or the avoidance of sexual intimacy (Parnham and Serefoglu, 2016).

Because many of the factors associated with sexual function and dysfunction are of genetic basis, this field has become a focus of attention for behavioral geneticists (Ciocanel et al., 2019). In the 1940s, Schapiro et al., have found that the incidences of PE in the male family members (fathers or brothers) of PE patients are higher than those without PE patients (Schapiro, 1943), which suggests that PE seems to be familial. This conclusion has also been confirmed by Waldinger et al. (1998). Additionally, Jern et al. (2007) conducted twin model-fitting analyses with different indicator variables of ejaculatory function based on a population sample of 3,946 twins and their siblings to investigate the genetic effects on PE and found a significant moderate genetic effect (28%) in PE, which provide a convincing proof for the above conclusion (Jern et al., 2007).

Notably, gene polymorphisms might play an important role in the etiology of LPE (Wang et al., 2021). From the recent study by Waldinger et al. (2011), we know that genetic factors might be associated with the pathogenesis of LPE. In other words, LPE might be related to some genetic factors, such as weakened central serotonin (5-HT) neurotransmission, hyperfunction of 5-HT1A receptors, and decreased 5-HT2C receptor function (Giuliano and Clément, 2006). Janssen et al. (2014) have found that 5-hydroxytryptamine transporter gene-linked polymorphic region (5-HTTLPR) polymorphism is associated with the duration of the intravaginal ejaculatory latency times (IELTs) by using a stopwatch in 89 Dutchmen with LPE. Among men who ejaculate within 1 min after vaginal penetration, men with LL genotype ejaculated 100% faster than men with SS genotype and 90% faster than men with SL genotype. In addition, findings from another study have shown that Cys23Ser 5-HT2C receptor gene polymorphism might be associated with the IELTs in the Dutch Caucasian men with LPE. Moreover, men with Cys/Cys genotypes are more likely to report shorter IELTs than men with Ser/Ser genotypes (Janssen et al., 2009).

However, the quest for identifying the actual genetic loci that contribute to LPE etiology has not been successful. Jannini et al. (2015) thought that candidate gene studies of PE are much like the candidate gene studies in other fields of research (Jannini et al., 2015), which is disappointing. Due to discrepancies in the design and methods of research, the correlation of most reports was not obtained in subjective replication experiments, and the conclusions may be inconsistent. In our study, three groups of ejaculation rats, namely, “rapid, normal, and delayed,” were selected based on the animal model of PE in rats and the theory of ejaculation. Among them, the rats in the “rapid” ejaculation group can be used to stimulate humans with PE. Subsequently, we used the rat brain tissue for whole-transcriptome sequencing to screen the differential genes among the three groups and provide a theoretical basis for the targeted therapy of PE.

## Materials and Methods

### Before Copulatory Test

#### Sprague-Dawley Rats Preparation

Experiments were carried out in the animal laboratory center of the Anhui Medical University after the approval of the Animal Ethics Committee of the Anhui Medical University, China.

Adult male and female SD rats (male: 250–350 g; female: 200–300 g) obtained from the Anhui Medical University Animal Experiment Center were used in all experiments. At the start of experiments, animals were housed under reversed 12-h dark/12-h light cycle conditions (light from 7:00 p.m. to 7:00 a.m.). Male and female rats were housed individually (with a controlled room temperature of 20 ± 2°C and relative humidity of 40–50%) and provided with free access to food and water. To adapt to the experimental environment, these animals should arrive at the laboratory at least 2 weeks before the start of the experiment. Female rats underwent ovariectomy and recovered at least 1 week before the copulatory test.

#### Materials

Drugs, such as estradiol benzoate (2 ml: 4 mg), progesterone injection (2 ml: 20 mg), and sesame oil, were used. A high-definition digital camera system (EZVIZ S2 GoPro), mobile hard disk drive, desk lamp, and surgical instruments were used. An observation cage was used with dimensions of 60 cm × 40 cm × 40 cm.

#### Inducing

Female rats were ovariectomized and recovered at least 1 week before the copulatory test. Estradiol benzoate (20 μg) and progesterone (500 μg) were dissolved in 0.1 ml sesame oil in a 60°C water bath. Then the obtained drugs were subcutaneously injected into the ovariectomized female rats 48 h and 4 h before the copulatory test, respectively. Stimulus female rats were used once a week, and female rats that did not show receptive and perceptive behavior during testing were replaced by others.

#### Copulatory Tests

A total of 105 male rats received six copulatory tests (15 were screened daily once a week for 6 consecutive weeks). The copulatory test was arranged from 7:00 p.m. to 9:00 p.m. Under the illumination of a dim warm lamp, each single male rat was put into the observation cage first. After 10 min of adaptation, a female rat in full estrus was inserted into the same cage allowing free copulation for 1 h.

Copulatory tests and sexual behavior were recorded using a camera (EZVIZ S2 motion camera), and the following parameters were counted:

Mount latency (ML): Latency until the first mount.
Mount frequency (MF): Number of mounts before the first ejaculation.
Intromission latency (IL): Latency until the first intromission.
Intromission frequency (IF): Number of intromissions before the first ejaculation.
Ejaculation latency (EL): Time from the first intromission until ejaculation.
Ejaculation frequency (EF): Number of ejaculation during 1 h.
Post-ejaculatory interval (PEI): Time from ejaculation until the first mount/intromission event of the next cycle.
Intromission rate (IR): IF/(IF + MF).

#### Male Rat Categorization Procedure

The behavioral experiment was conducted according to Pattij et al.’s methods (Pattij et al., 2005). Six copulatory tests were conducted for all observed male rats. To make the experimental data reliable, each male rat would be fixed with a steroid-stimulated female rat once a week in a cage for 1 h, and the male rats were put into the observation cage before 10 min to adapt to the environment.

Based on the “ejaculation distribution theory (EDT)” postulated by Waldinger et al. (2005), experimental animals were classified by their average EF (from four to sixth copulatory tests) as follows: (a) “sluggish group,” corresponding to 0–10% of the overall male population with the lowest EF (10th centile); (b) “normal group,” corresponding to the median ± 5% of the overall male population with the normal EF (45th to 55th centile); and (c) “rapid group,” corresponding to ∼10% of the overall male population with the highest EF (90th centile).

#### RNA Isolation and Preparation for RNA-Seq

Immediately after removal, the rat brain tissue was quickly frozen in liquid nitrogen and stored at −80°C until processing. Total RNA was isolated using Trizol reagent (Invitrogen, United States), following the manufacturer’s protocol.

The purity and concentration of total RNA were detected using agarose gel electrophoresis and DS-11 Spectrophotometer (DeNovix Corporation, United States) using rats’ RNA for sequencing in this experiment.

First, the MGIEasy rRNA removal kit was used to remove the ribosomal RNA from the extracted total RNA. After purification, the RNA was fragmented under a certain temperature and ionic environment. Then, the random primers and reverse transcriptase in the MGIEasy RNA directional library preparation kit were used to synthesize one-stranded cDNA, and DNA polymerase I and RNaseH were used to synthesize double-stranded cDNA. In the process of two-stranded synthesis of cDNA, the RNA template was removed and dTTP was replaced with dUTP. The participation of dUTP prevented the second strand of cDNA from being amplified in the subsequent process because the polymerase cannot cross the dUTP site on the template during extension. The double-stranded cDNA product was then ligated with an “A” base and a linker. The ligation product was amplified, and the PCR product was thermally denatured to single-stranded DNA, and then the single-stranded DNA was circularized with a bridge primer to obtain a single-stranded circular DNA library, which was sequenced on the computer.

#### Quantitative-PCR

The purity and concentration of total RNA were detected using agarose gel electrophoresis and DS-11 Spectrophotometer (DeNovix Corporation, United States) using rats’ RNA for sequencing in this experiment. Subsequently, cDNA synthesis was performed using a DNA Reverse Transcription Kit, and the levels of *BGIG10116_34868* mRNA were detected using Q-PCR. GAPDH was used as the internal reference for the *BGIG10116_34868* gene.

The following primers were used: *BGIG10116_34868*, forward 5′-CGCTTGTTCTCACCCAACAA-3′, and reverse 5′-GTGACTCGGCCCTAGGTATC-3′. In this study, Q-PCR was performed using Applied Biosystems TM 7500 instrument (Applied Biosystems, Inc) The reaction conditions for Q-PCR were set as follows: 95°C for 1 min for 1 cycle, and 95°C for 20 s, 60°C for 20 s, and 72°C for 30 s for 40 cycles. After Q-PCR detection, the levels of the *BGIG10116_34868* gene were calculated according to the 2^–ΔΔ^Ct method.

### Statistical Analysis

For data analysis, the SPSS 13.0 software (SPSS Inc., Chicago, IL, United States) was used. Data were represented as mean ± standard deviation (SD) or percentage. The normal distribution test was used to analyze the distribution of EF values in male rats. Comparison of the quantitative data was based on the ANOVA, SNK-q test, Kruskal-Wallis test, and the Chi-square test. The Pearson correlation coefficient was used to evaluate the correlation of sexual behavior parameters (EF and EL) and *BGIG10116_34868*. The value of *P* < 0.05 was considered to be statistically significant.

## Results

### Behavioral Characteristics

After 6 weeks of copulatory tests, a total of 15 male rats (14.29%, 15/105) were excluded (1 male rat died and 14 had no insertion behavior in all copulatory tests). In addition, the ejaculation frequency of 20 male rats (19.05%, 20/105) varied greatly among the last three copulatory tests, and they are also excluded.

**FIGURE 1:**
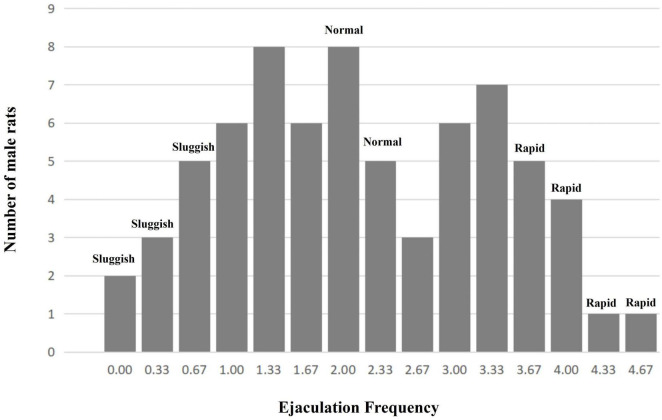
The number of premature ejaculation rats in different groups.

A total of 70 male rats’ copulatory tests were analyzed. Finally, 11 male rats were classified as the “rapid ejaculatory” group, 13 male rats were classified as the “normal ejaculatory” group, and 10 male rats were classified as the “sluggish ejaculatory” group (Figure 1). The parameters related to the sexual behavior of the three groups are listed in Table 1. Compared with the “normal ejaculatory” and “sluggish ejaculatory” groups, rats in the “rapid ejaculatory” group (reported highest EF) have shown shorter ML, MF, IL, EL, and IF.

**TABLE 1:** Sexual behavioral parameters of the male rats in different groups.

Behavioral parameters		Rapid	Normal	Sluggish	ANOVA
Mount	*ML (s)*	210.38 ± 148.83	363.79 ± 227.31	689.69 ± 376.30	0.001
	*MF (times)*	16.76 ± 5.13	34.44 ± 11.32	35.00 ± 16.49	0.001
Intromission	*IL (s)*	256.32 ± 169.98	513.69 ± 334.79	1087.18 ± 490.31	<0.001
	*IF (times)*	13.30 ± 3.54	24.64 ± 6.50	26.78 ± 13.11	0.001
Ejaculation	*EL (s)*	397.97 ± 164.02	1252.73 ± 528.80	2135.84 ± 263.87	< 0.001
	*EF (times)*	3.94 ± 0.33	2.13 ± 0.17	0.43 ± 0.27	<0.001
Other	*IR (IR* = *IF/[IF* + *EF])*	0.445 ± 0.069	0.423 ± 0.052	0.400 ± 0.046	0.243
	*PEI*	494.30 ± 42.30	478.54 ± 38.08	519.70 ± 122.42	0.438

*ML, mounting latency; MF, mounting frequency; IL, intromission latency; IF, intromission frequency; EL, ejaculation latency; EF, ejaculation frequency; IR, intromission rate; PEI, time from ejaculation until the first mount/intromission event of the next cycle.*

### Overview of the Landscape of the Rats’ Transcriptome

Long noncoding RNA (LncRNA) and smallRNA libraries were constructed from three groups of RNA samples derived from 15 male rats (five cases of “rapid,” “normal,” and “sluggish”). Bowtiwe2 (RC1) and RSEM (RC2) were used to calculate the expression of genes and transcripts.

A total of 38,490 RNAs were collected from the most authoritative databases, such as RefSeq, UCSC Genome Browser, Ensembl37.59, Reactome, and RNAdb2.0, as detailed in the manufacturer’s help information. These data were used to study the known data and rediscover potential biomarkers that have not been mined earlier.

**FIGURE 2:**
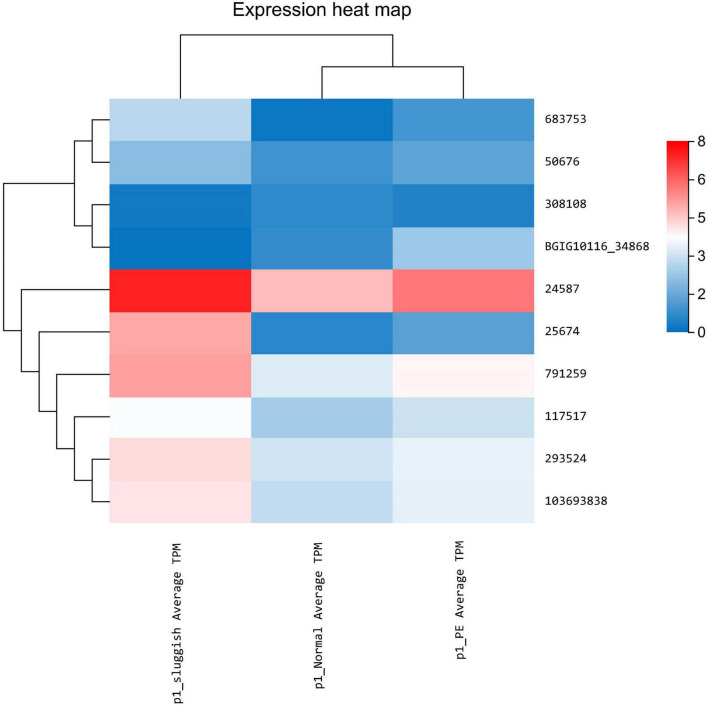
Expression of different genes in premature ejaculation grading.

**FIGURE 3:**
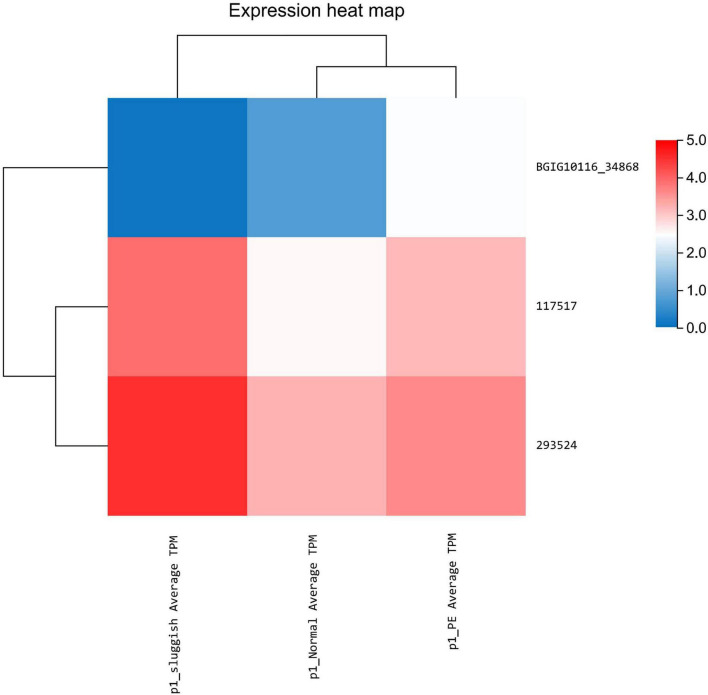
Expression of three key genes in premature ejaculation grading.

Differential expression of lncRNA was noted using statistical analysis for a total of 12 genes (10 upregulated and 2 downregulated) in the “normal vs. PE” group, 143 genes (123 upregulated and 20 downregulated) in the “normal vs. sluggish” group, and 9 genes (6 upregulated and 3 downregulated) in the “PE vs. sluggish” group. Because there were many different genes, we have listed only the genes with the most significant differences in expression (Figure 2). There were statistically significant differences in the expression of 3 genes (i.e., *BGIG10116_34868*, BAG3, and C7) between the “rapid,” “normal,” and “sluggish” groups (*P* < 0.05). Although more stringent screening criteria (*P* < 0.01) were used in our investigation, we found that one unknown candidate gene *BGIG10116_34868* had significant differences among the 3 groups (“rapid,” “normal,” and “sluggish”), and the gene expression was highest in the “rapid” group and lowest in the “normal” group (Figure 3).

**FIGURE 4:**
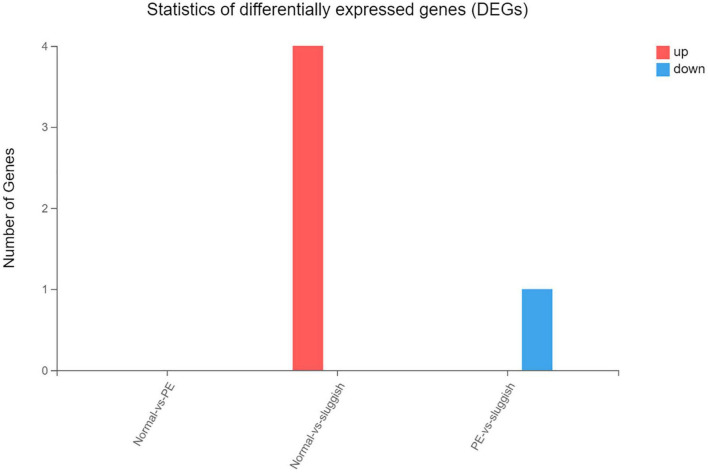
The number of up-regulated and down-regulated genes in the comparison of different grades.

However, there was no significant difference in the smallRNA gene expression among the three groups. Comparing the “normal” group and the “sluggish” group, there were 4 genes that were upregulated. Comparing the “rapid” group and the “sluggish” group, there was only 1 gene that was downregulated (Figure 4).

**FIGURE 5:**
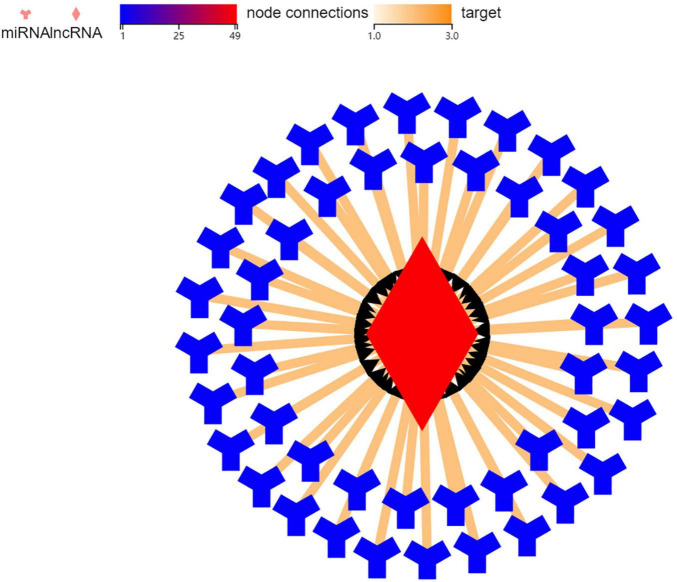
Correlation between LncRNA and miRNA.

In order to explore the mechanism of *BGIG10116_34868* action 49 genes were related to it through targeted prediction, which included miR-506, miR-105, miR-271, and miR-178 (Figure 5).

### Results of Q-PCR

**FIGURE 6:**
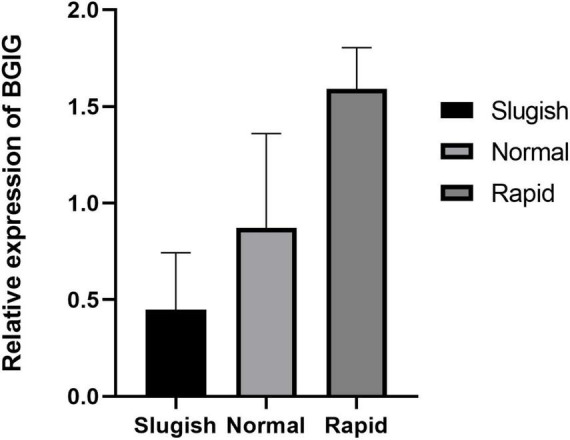
The relative expression of BGIG in different premature ejaculation grades.

PCR analysis was used to validate the changes in the expression of the genes identified. The results of the expression analysis revealed similar trends in the expression of the gene (BGIG10116-34868) compared with the statistically significant sequencing results (*P* < 0.05) (Figure 6).

### Correlation Between BGIG10116-34868 and Copulatory Behavior

In copulatory tests, EF and EL were the two main indicators of ejaculation ability, so we selected them for correlation analysis. The correlation between the sexual behavioral parameters (including EF and EL) and BGIG10116-34868 in the male rats is shown in Table 2. Three of the results were positively correlated with EF (*r* = 0.653, *P* < 0.05). However, a negative correlation between EL and BGIG10116-34868 was found in male rats (*r* = -0.606, *P* < 0.05).

**TABLE 2:** Correlation between relative BGIG expression and copulatory behavior.

	EF		EL	
	*r*	*p*	*r*	*p*
Relative BGIG Expression	0.653	<0.05	−0.606	< 0.05

*EF, ejaculation frequency; EL, ejaculation latency.*

## Discussion

For human beings, ejaculation is considered one of the most amazing living phenomena in the world of biology (Giuliano, 2011). PE, ejaculation retardation, and other ejaculation dysfunction not only affect fertility but also affect the quality of sexual life (Burri et al., 2018). Therefore, the study on the molecular mechanism of brain neuroregulation during animal ejaculation will not only open up new ideas for understanding the physiological rules of animal ejaculation but also provide a new scientific basis for clinical research.

In this study, differential expression was noted using statistical analysis for a total of 12 genes (10 upregulated and 2 downregulated) in the “normal vs. PE” group, 143 genes (123 upregulated and 20 downregulated) in the “normal vs. sluggish” group, and 9 genes (6 upregulated and 3 downregulated) in the “PE vs. sluggish” group by sequencing the brain tissue of male rats in three different ejaculatory states. Further analysis showed that three genes (i.e., *BGIG10116_34868*, BAG3, and C7) differed between the three groups (*P* < 0.05). Notably, BGIG10116-34868 had significant differences among the 3 groups (*P* < 0.01), which has not been found earlier. To verify the accuracy of sequencing, we repeated experiments using Q-PCR, and the result was consistent with sequencing.

Further analysis of the association between lncRNA (*BGIG10116_34868*) and ejaculation showed that BGIG10116-34868 was correlated with EF and EL, which suggested that genetic changes may affect the ejaculation behavior.

In other animal experiments, studies have shown that after 8-OH-DPAT and dapoxetine were administered to rats in three different ejaculatory states, a large number of genes in the central nervous system could be found upregulated or downregulated, and their ejaculatory parameters also changed (Qin et al., 2017b). Santtila et al. (2010) found that carriers of the 10R10R genotype had lower scores compared with the combined 9R9R/9R10R carrier group by analyzing dopamine (DA)-transporter gene (DAT1) and four indicators (i.e., anteportal ejaculation, number of penile thrusts, ejaculation latency time, and feeling of control over ejaculation) associated with PE function, for anteportal ejaculation, number of thrusts, and feeling of control over ejaculation had a significant difference, except for ejaculation latency time.

To explore the mechanism of BGIG10116-34868, we carried out targeted predictions and found that genes, such as miR-506 and miR-105, were related. According to previous studies, miR-506 and miR-105 played a certain role in regulating the 5-HT system and the DA system. MiR-506 was a component of a cluster of X-chromosomes linked with miRNA (Yoshida et al., 2021), which had been reported to function as an important tumor suppressor in many human cancers. Peng et al. (2016) found that miR-506 acts as a tumor-suppressive agent by targeting signal transducer and activator of transcription 3 (STAT3), indicating that STAT3 was a direct target of miR-506. In addition, a study found that pharmacological inhibition of STAT3 increased the serotonin transporter (SERT) expression (also known as 5-HT transporter (5-HTT)), thus reducing depression-like behavior in wild-type rats in a depressed mouse model (Kong et al., 2015). The neurobiology of ejaculation was complex with the 5-HT transporter system playing a central role (Xia et al., 2020). Polymorphism in SLC6A4 encodes a 5-HT transporter (5-HTT) which is a major regulator of serotonin neurotransmission and is involved in the pathogenesis of PE. 5-HTT is responsible for clearing 5-HT from synapses and thus acts as a regulator of presynaptic and postsynaptic 5-HT receptor stimulation. Obviously, the change in SERT/5-HTT also has a certain effect on PE (Ye et al., 2020). As a result of all the above factors combined, we can speculate that miR-506 can regulate ejaculation function by STAT targeting SERT/5-HTT.

MiR-105 belongs to the GABRA3A gene region on the X chromosome. LncRNA has been shown to modulate the expression of DA, which is another major central neurotransmitter in psychiatric disorders. Zhao et al. (2017) reported that chronic morphine can induce a sustained increase in the expression of the D1 receptor at the glutamic-ergic terminal of projection neurons from the medial prefrontal cortex (mPFC) to the basolateral amygdala (BLA), due to downregulation of miR-105. Evidence of DA hinting to regulate male sexual behavior has been reported since the early 1970s. Studies have shown that systemic injection of selective D1-like antagonist SCH23390 can reduce the number of ejaculations before ejaculation and during the mating test in male rats (Domínguez-Salazar et al., 2014). In recent years, studies have shown that DA promotes semen excretion/ejaculation through the D2 receptor (Hyun et al., 2002). Therefore, both the DA and 5-HT systems are indispensable during ejaculation and appear to regulate sexual behavior in both men and women. DA seems to be the “trigger” for sex, while 5-HT acts as the “brake” (Qin et al., 2017a).

The etiology and pathogenesis of ejaculation dysfunction disorder are still not clear, and the genetic factors are considered to be closely related (Mostafa et al., 2020). In this context, we identified a novel gene, namely, BGIG10116-34868, by sequencing rat brain tissue, which may be a therapeutic target for PE. As a new gene, the study of this gene is still in the primary stage and there are many subsequent studies that should be needed, such as knocking out the BGIG gene of rats to observe their sexual behavior.

## Conclusion

By sequencing rat brain tissue, we first discovered that only one unknown candidate gene, namely, BGIG10116-34868 has significant differences among the 3 groups (“rapid,” “normal,” and “sluggish”). BGIG10116-34868 is also linearly correlated with EF and EL. As a new gene, BGIG10116-34868 may be a potential target gene for PE, which provides a new direction for future research on ejaculation dysfunction.

## Data Availability Statement

The datasets presented in this study can be found in online repositories. The names of the repository/repositories and accession number(s) can be found below: https://www.ncbi.nlm.nih.gov/bioproject/PRJNA760464.

## Ethics Statement

The animal study was reviewed and approved by Anhui Medical University LLSC20211063.

## Author Contributions

JG, HJ, and XZ conceived and designed the study, and revised the article for intellectual content. JG, RG, HL, XL, and PG acquired the data and analyzed and interpreted the data. JG, RG, PG, HL, XL, and JD drafted the article. All authors contributed to the study’s conception and design, read, and approved the final manuscript.

## Conflict of Interest

The authors declare that the research was conducted in the absence of any commercial or financial relationships that could be construed as a potential conflict of interest.

## Publisher’s Note

All claims expressed in this article are solely those of the authors and do not necessarily represent those of their affiliated organizations, or those of the publisher, the editors and the reviewers. Any product that may be evaluated in this article, or claim that may be made by its manufacturer, is not guaranteed or endorsed by the publisher.
